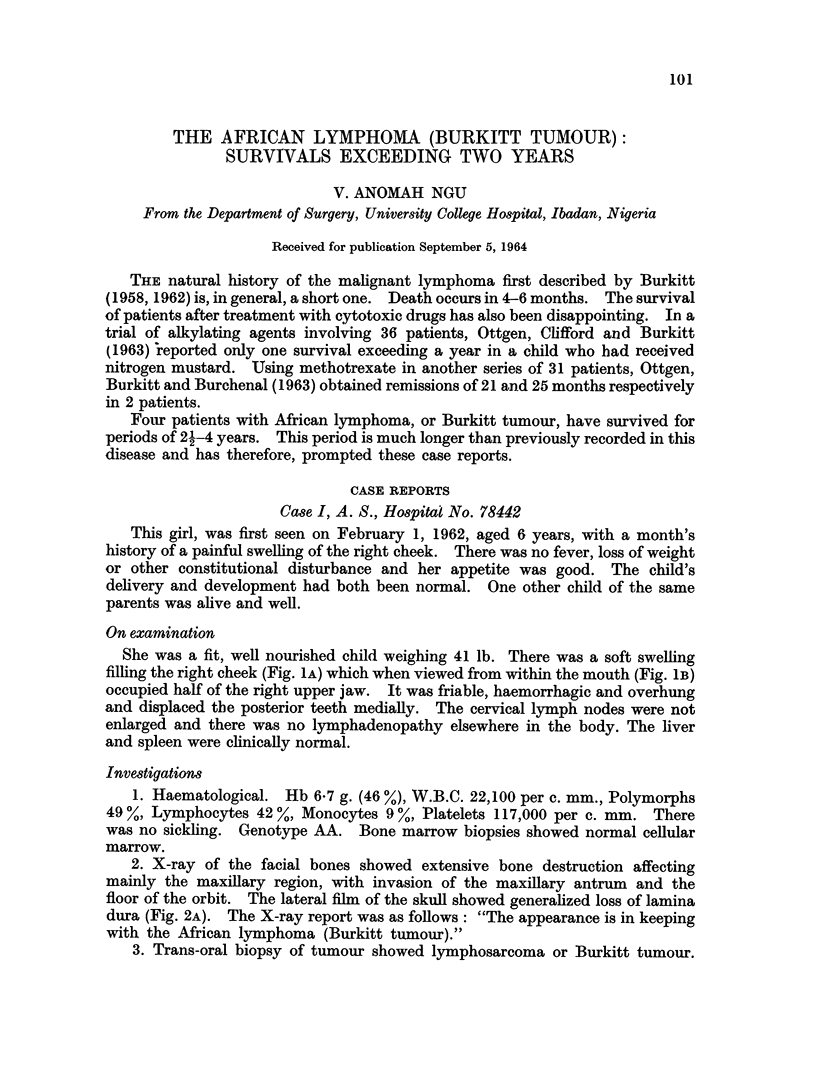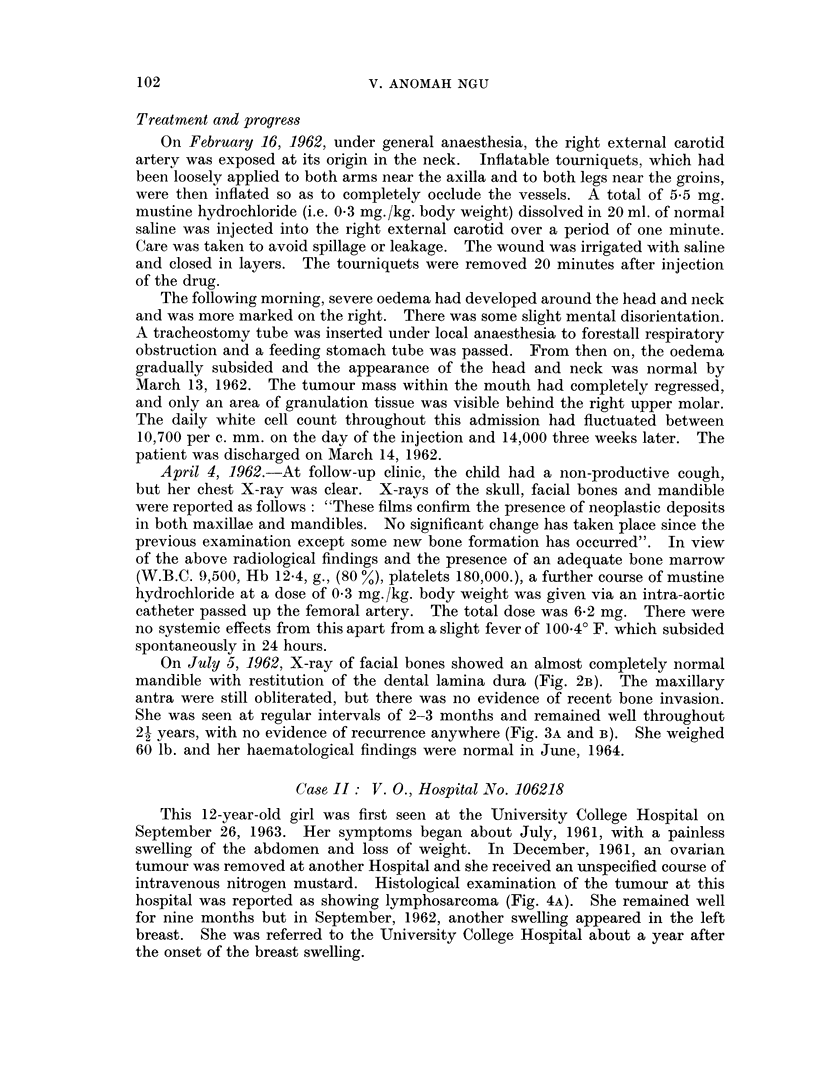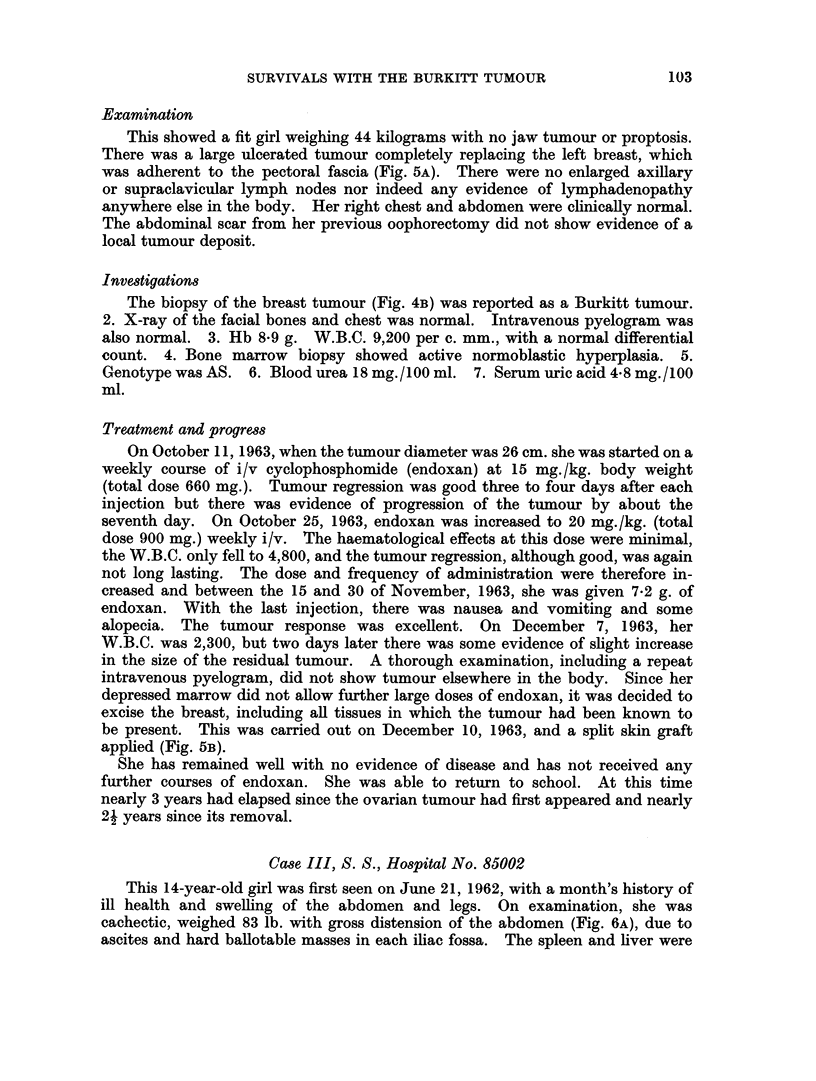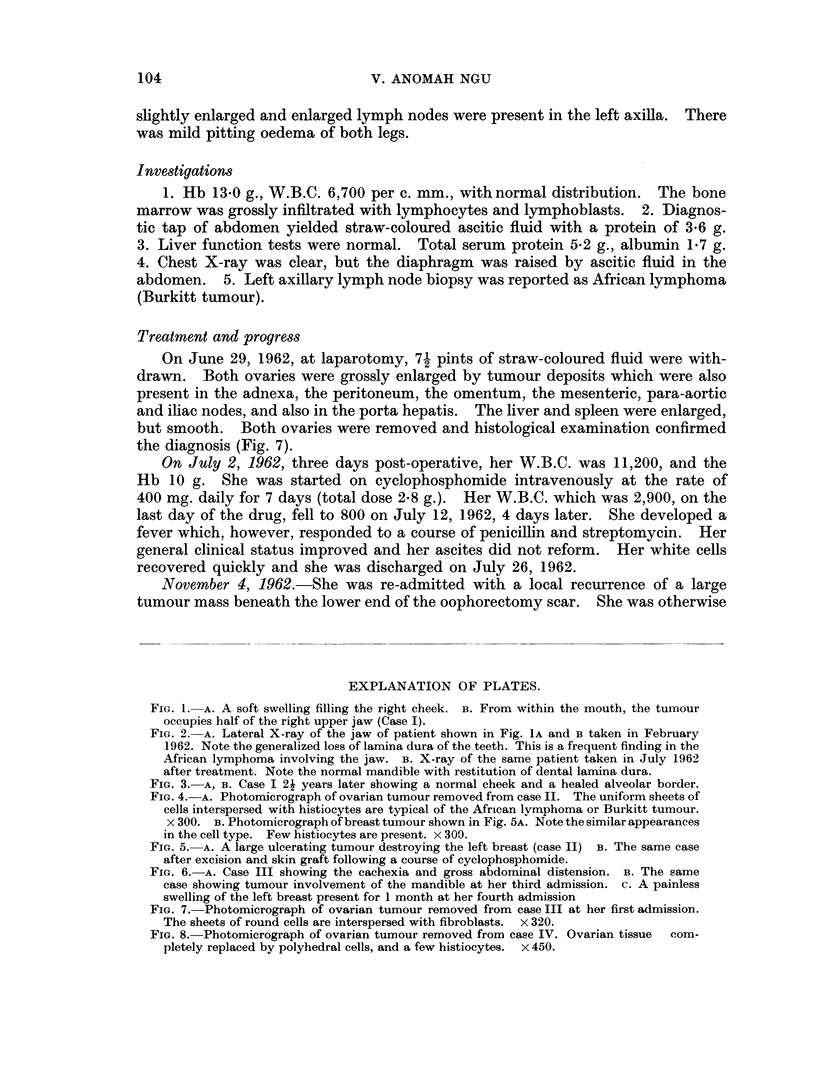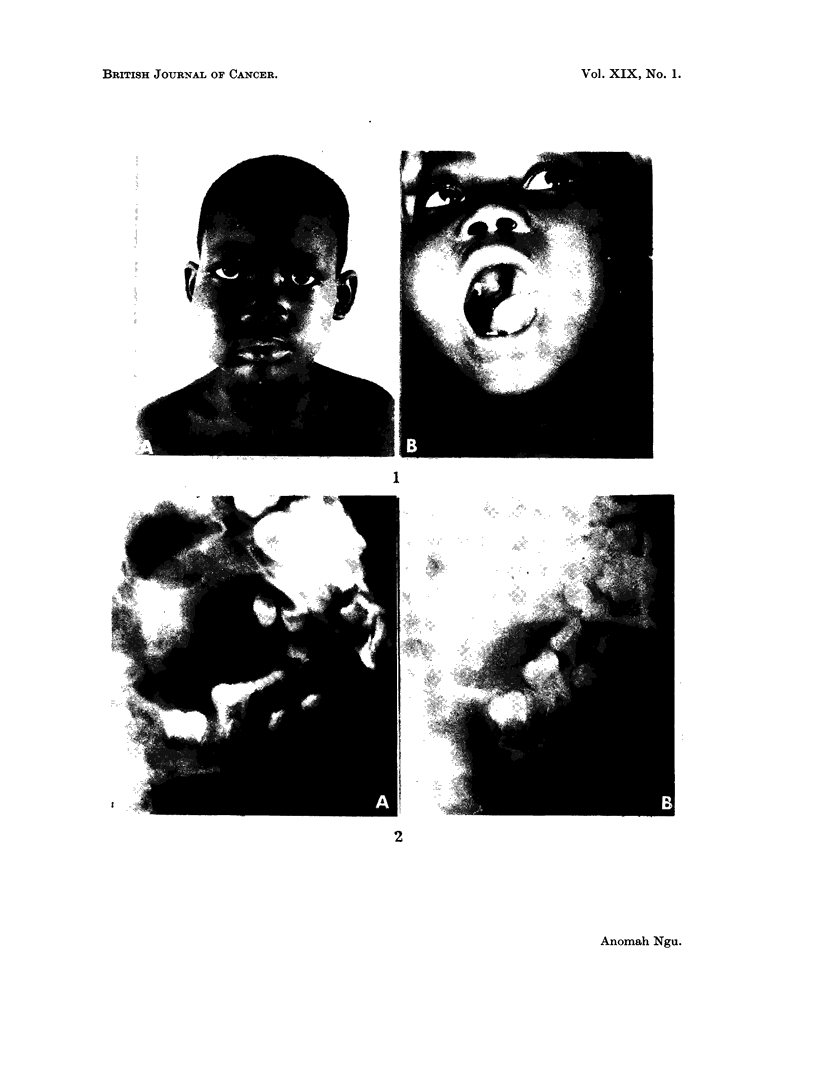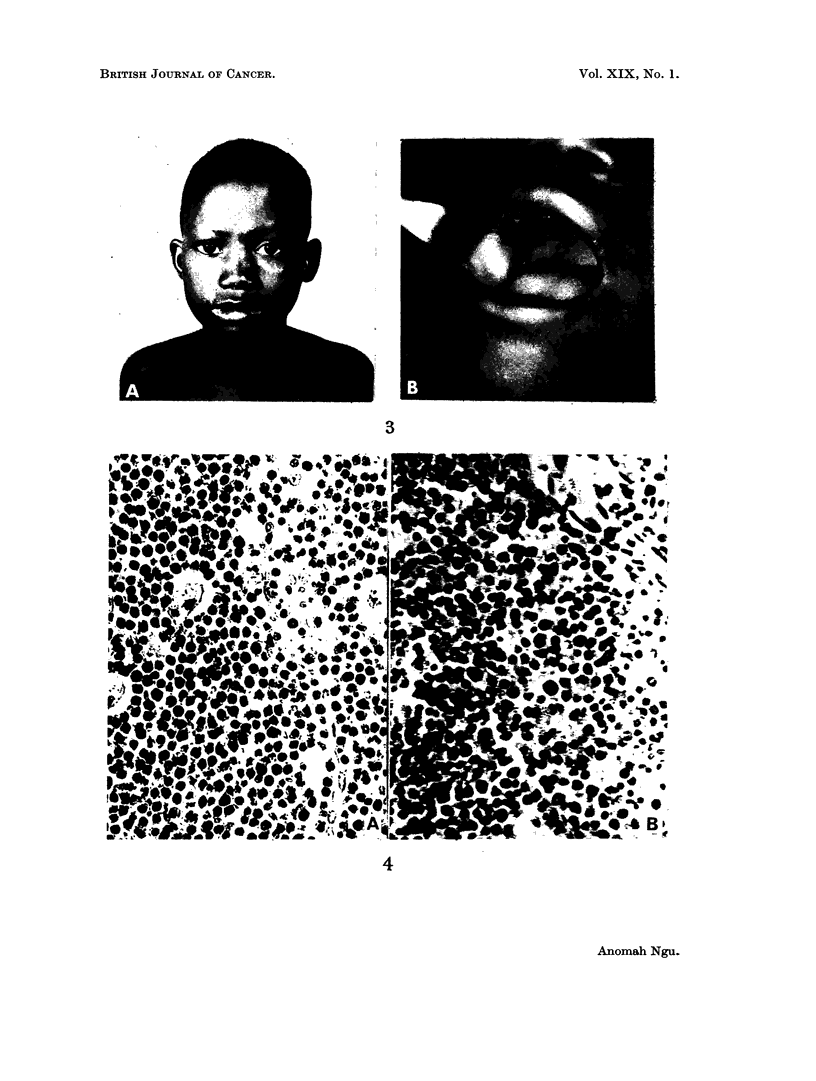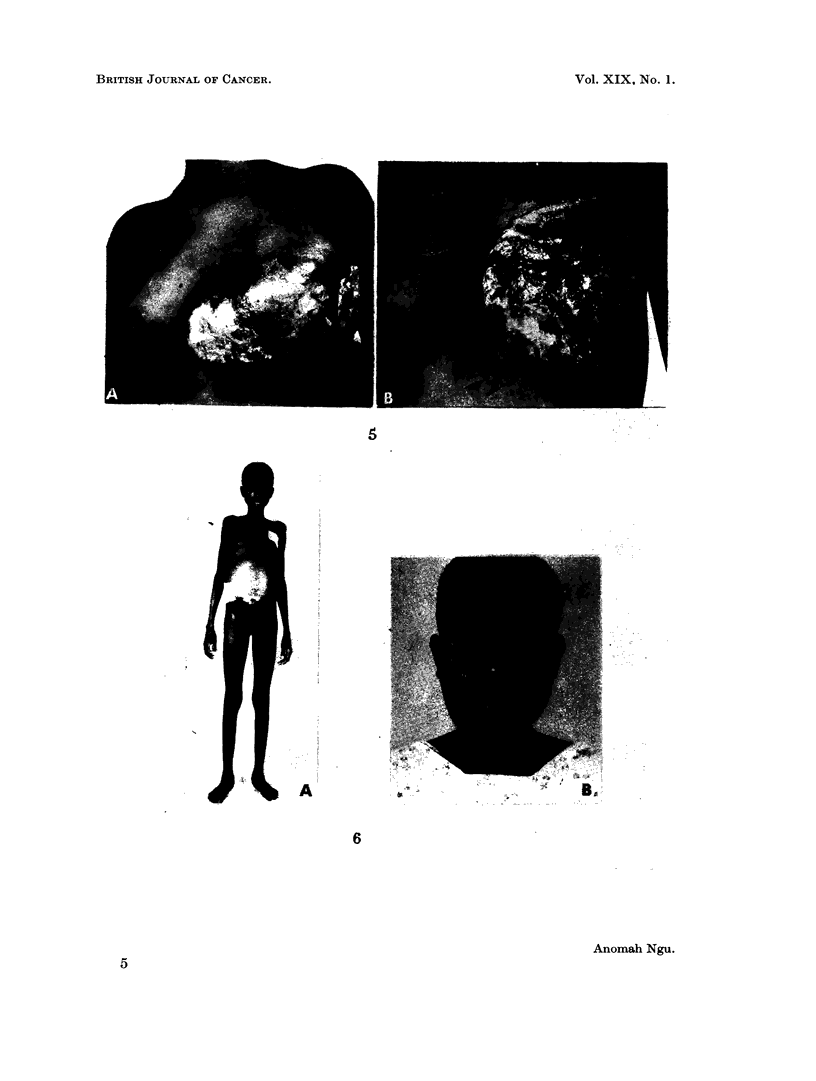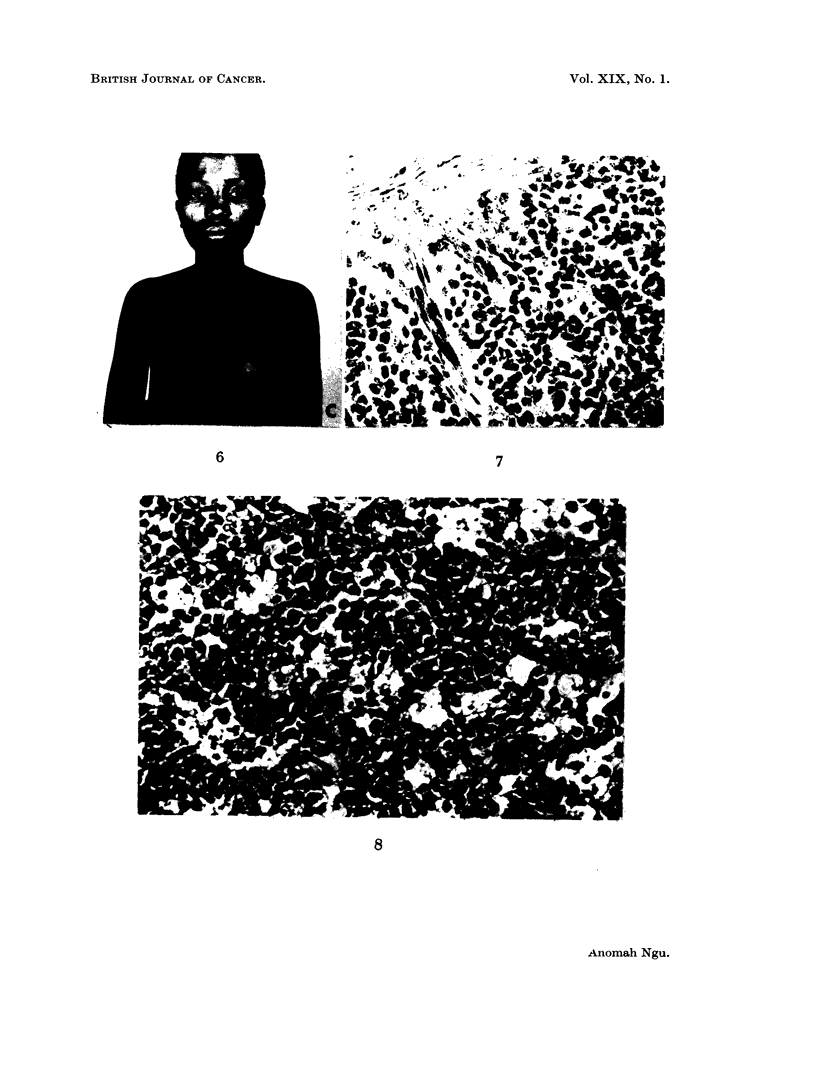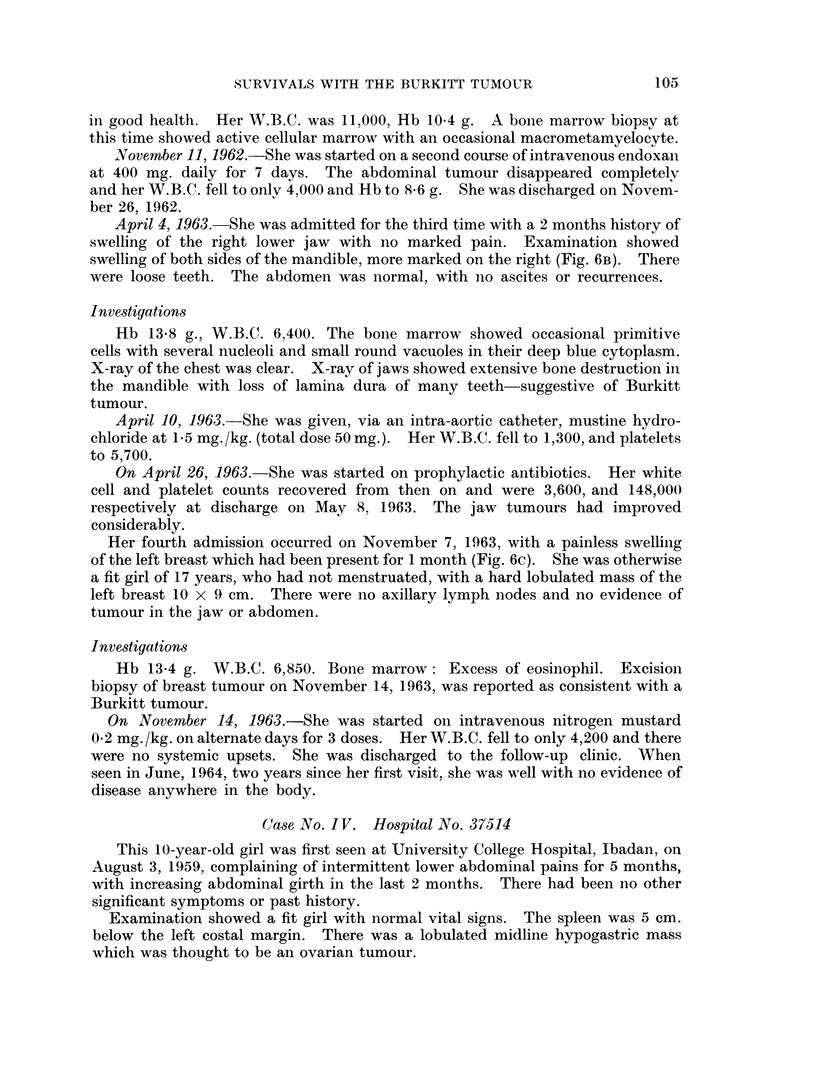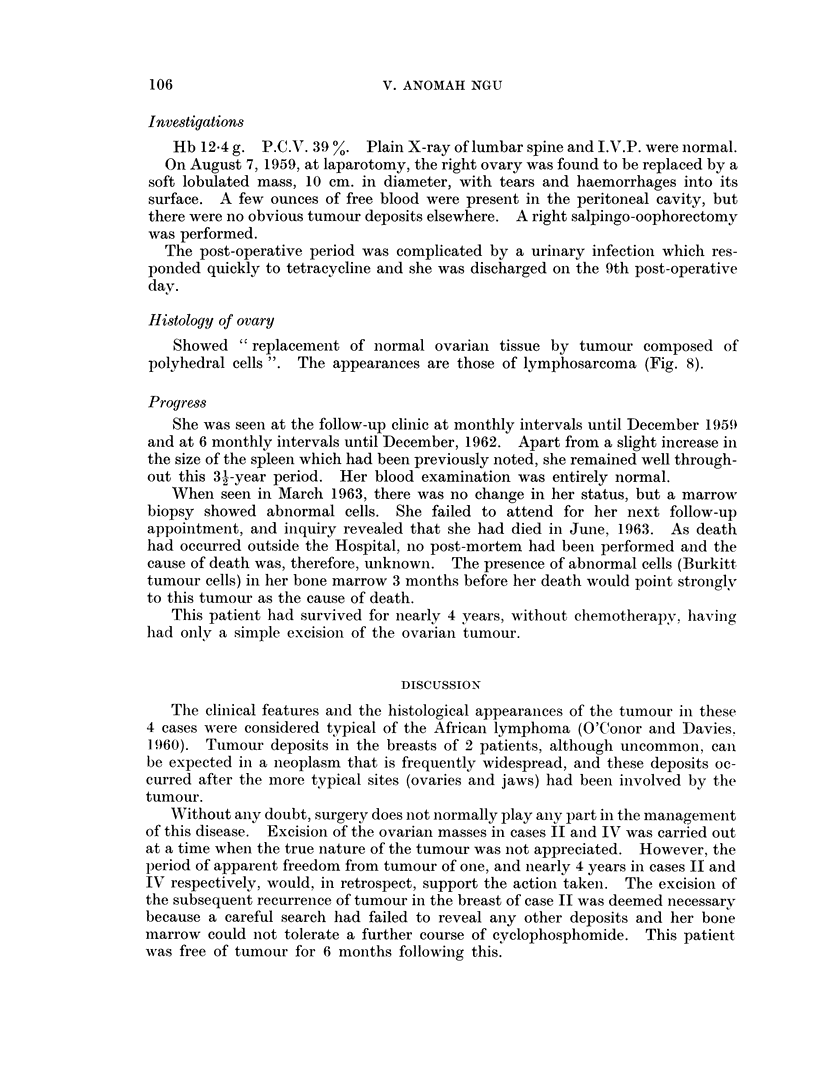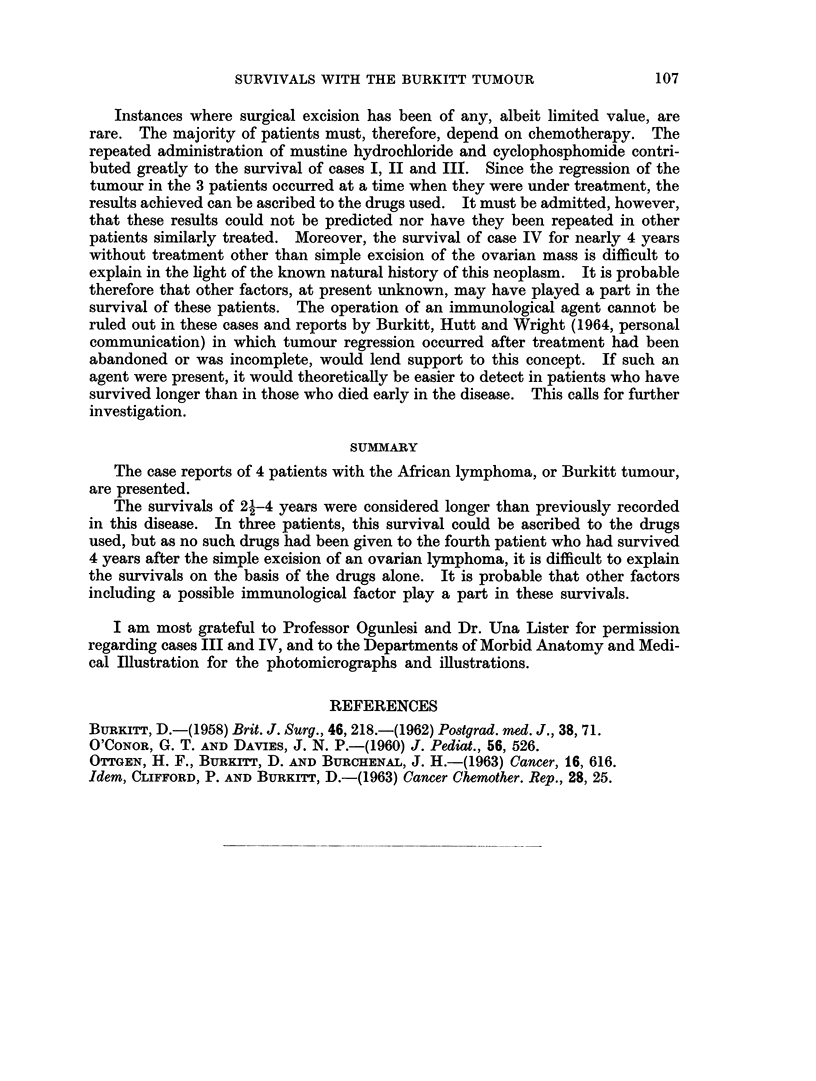# The African Lymphoma (Burkitt Tumour): Survivals Exceeding Two Years

**DOI:** 10.1038/bjc.1965.10

**Published:** 1965-03

**Authors:** V. Anomah Ngu

## Abstract

**Images:**


### THE AFRICAN LYMPHOMA (BURKITT TUMOUR): SURVIVALS EXCEEDING TWO YEARS

V. ANOMAH NGU

*From the Department of Surgery, University College Hospital, Ibadan, Nigeria*

Received for publication September 5, 1964

THE natural history of the malignant lymphoma first described by Burkitt (1958, 1962) is, in general, a short one. Death occurs in 4–6 months. The survival of patients after treatment with cytotoxic drugs has also been disappointing. In a trial of alkylating agents involving 36 patients, Ottgen, Clifford and Burkitt (1963) reported only one survival exceeding a year in a child who had received nitrogen mustard. Using methotrexate in another series of 31 patients, Ottgen, Burkitt and Burchenal (1963) obtained remissions of 21 and 25 months respectively in 2 patients.

Four patients with African lymphoma, or Burkitt tumour, have survived for periods of 2½–4 years. This period is much longer than previously recorded in this disease and has therefore, prompted these case reports.

### CASE REPORTS

### *Case I, A. S., Hospital No. 78442*

This girl, was first seen on February 1, 1962, aged 6 years, with a month's history of a painful swelling of the right cheek. There was no fever, loss of weight or other constitutional disturbance and her appetite was good. The child's delivery and development had both been normal. One other child of the same parents was alive and well.

*On examination*

She was a fit, well nourished child weighing 41 lb. There was a soft swelling filling the right cheek (Fig. 1A) which when viewed from within the mouth (Fig. 1B) occupied half of the right upper jaw. It was friable, haemorrhagic and overhung and displaced the posterior teeth medially. The cervical lymph nodes were not enlarged and there was no lymphadenopathy elsewhere in the body. The liver and spleen were clinically normal.

*Investigations*

1. Haematological. Hb 6·7 g. (46 %), W.B.C. 22,100 per c. mm., Polymorphs 49 %, Lymphocytes 42 %, Monocytes 9 %, Platelets 117,000 per c. mm. There was no sickling. Genotype AA. Bone marrow biopsies showed normal cellular marrow.
2. X-ray of the facial bones showed extensive bone destruction affecting mainly the maxillary region, with invasion of the maxillary antrum and the floor of the orbit. The lateral film of the skull showed generalized loss of lamina dura (Fig. 2A). The X-ray report was as follows : "The appearance is in keeping with the African lymphoma (Burkitt tumour)."
3. Trans-oral biopsy of tumour showed lymphosarcoma or Burkitt tumour.

*Treatment and progress*

On *February 16, 1962*, under general anaesthesia, the right external carotid artery was exposed at its origin in the neck. Inflatable tourniquets, which had been loosely applied to both arms near the axilla and to both legs near the groins, were then inflated so as to completely occlude the vessels. A total of 5·5 mg. mustine hydrochloride (i.e. 0·3 mg./kg. body weight) dissolved in 20 ml. of normal saline was injected into the right external carotid over a period of one minute. Care was taken to avoid spillage or leakage. The wound was irrigated with saline and closed in layers. The tourniquets were removed 20 minutes after injection of the drug.

The following morning, severe oedema had developed around the head and neck and was more marked on the right. There was some slight mental disorientation. A tracheostomy tube was inserted under local anaesthesia to forestall respiratory obstruction and a feeding stomach tube was passed. From then on, the oedema gradually subsided and the appearance of the head and neck was normal by March 13, 1962. The tumour mass within the mouth had completely regressed, and only an area of granulation tissue was visible behind the right upper molar. The daily white cell count throughout this admission had fluctuated between 10,700 per c. mm. on the day of the injection and 14,000 three weeks later. The patient was discharged on March 14, 1962.

*April 4, 1962.*—At follow-up clinic, the child had a non-productive cough, but her chest X-ray was clear. X-rays of the skull, facial bones and mandible were reported as follows : "These films confirm the presence of neoplastic deposits in both maxillae and mandibles. No significant change has taken place since the previous examination except some new bone formation has occurred". In view of the above radiological findings and the presence of an adequate bone marrow (W.B.C. 9,500, Hb 12·4, g., (80 %), platelets 180,000.), a further course of mustine hydrochloride at a dose of 0·3 mg./kg. body weight was given via an intra-aortic catheter passed up the femoral artery. The total dose was 6·2 mg. There were no systemic effects from this apart from a slight fever of 100·4° F. which subsided spontaneously in 24 hours.

On *July 5, 1962*, X-ray of facial bones showed an almost completely normal mandible with restitution of the dental lamina dura (Fig. 2B). The maxillary antra were still obliterated, but there was no evidence of recent bone invasion. She was seen at regular intervals of 2–3 months and remained well throughout 2½ years, with no evidence of recurrence anywhere (Fig. 3A and B). She weighed 60 lb. and her haematological findings were normal in June, 1964.

*Case II : V. O., Hospital No. 106218*

This 12-year-old girl was first seen at the University College Hospital on September 26, 1963. Her symptoms began about July, 1961, with a painless swelling of the abdomen and loss of weight. In December, 1961, an ovarian tumour was removed at another Hospital and she received an unspecified course of intravenous nitrogen mustard. Histological examination of the tumour at this hospital was reported as showing lymphosarcoma (Fig. 4A). She remained well for nine months but in September, 1962, another swelling appeared in the left breast. She was referred to the University College Hospital about a year after the onset of the breast swelling.

*Examination*

This showed a fit girl weighing 44 kilograms with no jaw tumour or proptosis. There was a large ulcerated tumour completely replacing the left breast, which was adherent to the pectoral fascia (Fig. 5A). There were no enlarged axillary or supraclavicular lymph nodes nor indeed any evidence of lymphadenopathy anywhere else in the body. Her right chest and abdomen were clinically normal. The abdominal scar from her previous oophorectomy did not show evidence of a local tumour deposit.

*Investigations*

The biopsy of the breast tumour (Fig. 4B) was reported as a Burkitt tumour. 2. X-ray of the facial bones and chest was normal. Intravenous pyelogram was also normal. 3. Hb 8·9 g. W.B.C. 9,200 per c. mm., with a normal differential count. 4. Bone marrow biopsy showed active normoblastic hyperplasia. 5. Genotype was AS. 6. Blood urea 18 mg./100 ml. 7. Serum uric acid 4·8 mg./100 ml.

*Treatment and progress*

On October 11, 1963, when the tumour diameter was 26 cm. she was started on a weekly course of i/v cyclophosphomide (endoxan) at 15 mg./kg. body weight (total dose 660 mg.). Tumour regression was good three to four days after each injection but there was evidence of progression of the tumour by about the seventh day. On October 25, 1963, endoxan was increased to 20 mg./kg. (total dose 900 mg.) weekly i/v. The haematological effects at this dose were minimal, the W.B.C. only fell to 4,800, and the tumour regression, although good, was again not long lasting. The dose and frequency of administration were therefore increased and between the 15 and 30 of November, 1963, she was given 7·2 g. of endoxan. With the last injection, there was nausea and vomiting and some alopecia. The tumour response was excellent. On December 7, 1963, her W.B.C. was 2,300, but two days later there was some evidence of slight increase in the size of the residual tumour. A thorough examination, including a repeat intravenous pyelogram, did not show tumour elsewhere in the body. Since her depressed marrow did not allow further large doses of endoxan, it was decided to excise the breast, including all tissues in which the tumour had been known to be present. This was carried out on December 10, 1963, and a split skin graft applied (Fig. 5B).

She has remained well with no evidence of disease and has not received any further courses of endoxan. She was able to return to school. At this time nearly 3 years had elapsed since the ovarian tumour had first appeared and nearly 2½ years since its removal.

### *Case III, S. S., Hospital No. 85002*

This 14-year-old girl was first seen on June 21, 1962, with a month's history of ill health and swelling of the abdomen and legs. On examination, she was cachectic, weighed 83 lb. with gross distension of the abdomen (Fig. 6A), due to ascites and hard ballotable masses in each iliac fossa. The spleen and liver were slightly enlarged and enlarged lymph nodes were present in the left axilla. There was mild pitting oedema of both legs.

*Investigations*

1. Hb 13·0 g., W.B.C. 6,700 per c. mm., with normal distribution. The bone marrow was grossly infiltrated with lymphocytes and lymphoblasts. 2. Diagnostic tap of abdomen yielded straw-coloured ascitic fluid with a protein of 3·6 g. 3. Liver function tests were normal. Total serum protein 5·2 g., albumin 1·7 g. 4. Chest X-ray was clear, but the diaphragm was raised by ascitic fluid in the abdomen. 5. Left axillary lymph node biopsy was reported as African lymphoma (Burkitt tumour).

*Treatment and progress*

On June 29, 1962, at laparotomy, 7½ pints of straw-coloured fluid were withdrawn. Both ovaries were grossly enlarged by tumour deposits which were also present in the adnexa, the peritoneum, the omentum, the mesenteric, para-aortic and iliac nodes, and also in the porta hepatis. The liver and spleen were enlarged, but smooth. Both ovaries were removed and histological examination confirmed the diagnosis (Fig. 7).

*On July 2, 1962*, three days post-operative, her W.B.C. was 11,200, and the Hb 10 g. She was started on cyclophosphomide intravenously at the rate of 400 mg. daily for 7 days (total dose 2·8 g.). Her W.B.C. which was 2,900, on the last day of the drug, fell to 800 on July 12, 1962, 4 days later. She developed a fever which, however, responded to a course of penicillin and streptomycin. Her general clinical status improved and her ascites did not reform. Her white cells recovered quickly and she was discharged on July 26, 1962.

*November 4, 1962.*—She was re-admitted with a local recurrence of a large tumour mass beneath the lower end of the oophorectomy scar. She was otherwise

---

EXPLANATION OF PLATES.

FIG. 1.—A. A soft swelling filling the right cheek. B. From within the mouth, the tumour occupies half of the right upper jaw (Case I).

FIG. 2.—A. Lateral X-ray of the jaw of patient shown in Fig. 1A and B taken in February 1962. Note the generalized loss of lamina dura of the teeth. This is a frequent finding in the African lymphoma involving the jaw. B. X-ray of the same patient taken in July 1962 after treatment. Note the normal mandible with restitution of dental lamina dura.

FIG. 3.—A, B. Case I 2½ years later showing a normal cheek and a healed alveolar border.

FIG. 4.—A. Photomicrograph of ovarian tumour removed from case II. The uniform sheets of cells interspersed with histiocytes are typical of the African lymphoma or Burkitt tumour. ×300. B. Photomicrograph of breast tumour shown in Fig. 5A. Note the similar appearances in the cell type. Few histiocytes are present. ×300.

FIG. 5.—A. A large ulcerating tumour destroying the left breast (case II) B. The same case after excision and skin graft following a course of cyclophosphomide.

FIG. 6.—A. Case III showing the cachexia and gross abdominal distension. B. The same case showing tumour involvement of the mandible at her third admission. C. A painless swelling of the left breast present for 1 month at her fourth admission

FIG. 7.—Photomicrograph of ovarian tumour removed from case III at her first admission. The sheets of round cells are interspersed with fibroblasts. ×320.

FIG. 8.—Photomicrograph of ovarian tumour removed from case IV. Ovarian tissue completely replaced by polyhedral cells, and a few histiocytes. ×450.

1

2

Anomah Ngu.

3

4

Anomah Ngu.

5

6

6

7

8

in good health. Her W.B.C. was 11,000, Hb 10·4 g. A bone marrow biopsy at this time showed active cellular marrow with an occasional macrometamyelocyte.

*November 11, 1962.*—She was started on a second course of intravenous endoxan at 400 mg. daily for 7 days. The abdominal tumour disappeared completely and her W.B.C. fell to only 4,000 and Hb to 8·6 g. She was discharged on November 26, 1962.

*April 4, 1963.*—She was admitted for the third time with a 2 months history of swelling of the right lower jaw with no marked pain. Examination showed swelling of both sides of the mandible, more marked on the right (Fig. 6B). There were loose teeth. The abdomen was normal, with no ascites or recurrences.

*Investigations*

Hb 13·8 g., W.B.C. 6,400. The bone marrow showed occasional primitive cells with several nucleoli and small round vacuoles in their deep blue cytoplasm. X-ray of the chest was clear. X-ray of jaws showed extensive bone destruction in the mandible with loss of lamina dura of many teeth—suggestive of Burkitt tumour.

*April 10, 1963.*—She was given, via an intra-aortic catheter, mustine hydrochloride at 1·5 mg./kg. (total dose 50 mg.). Her W.B.C. fell to 1,300, and platelets to 5,700.

*On April 26, 1963.*—She was started on prophylactic antibiotics. Her white cell and platelet counts recovered from then on and were 3,600, and 148,000 respectively at discharge on May 8, 1963. The jaw tumours had improved considerably.

Her fourth admission occurred on November 7, 1963, with a painless swelling of the left breast which had been present for 1 month (Fig. 6C). She was otherwise a fit girl of 17 years, who had not menstruated, with a hard lobulated mass of the left breast 10 × 9 cm. There were no axillary lymph nodes and no evidence of tumour in the jaw or abdomen.

*Investigations*

Hb 13·4 g. W.B.C. 6,850. Bone marrow : Excess of eosinophil. Excision biopsy of breast tumour on November 14, 1963, was reported as consistent with a Burkitt tumour.

*On November 14, 1963.*—She was started on intravenous nitrogen mustard 0·2 mg./kg. on alternate days for 3 doses. Her W.B.C. fell to only 4,200 and there were no systemic upsets. She was discharged to the follow-up clinic. When seen in June, 1964, two years since her first visit, she was well with no evidence of disease anywhere in the body.

### *Case No. IV. Hospital No. 37514*

This 10-year-old girl was first seen at University College Hospital, Ibadan, on August 3, 1959, complaining of intermittent lower abdominal pains for 5 months, with increasing abdominal girth in the last 2 months. There had been no other significant symptoms or past history.

Examination showed a fit girl with normal vital signs. The spleen was 5 cm. below the left costal margin. There was a lobulated midline hypogastric mass which was thought to be an ovarian tumour.

*Investigations*

Hb 12·4 g. P.C.V. 39 %. Plain X-ray of lumbar spine and I.V.P. were normal.

On August 7, 1959, at laparotomy, the right ovary was found to be replaced by a soft lobulated mass, 10 cm. in diameter, with tears and haemorrhages into its surface. A few ounces of free blood were present in the peritoneal cavity, but there were no obvious tumour deposits elsewhere. A right salpingo-oophorectomy was performed.

The post-operative period was complicated by a urinary infection which responded quickly to tetracycline and she was discharged on the 9th post-operative day.

*Histology of ovary*

Showed " replacement of normal ovarian tissue by tumour composed of polyhedral cells ". The appearances are those of lymphosarcoma (Fig. 8).

*Progress*

She was seen at the follow-up clinic at monthly intervals until December 1959 and at 6 monthly intervals until December, 1962. Apart from a slight increase in the size of the spleen which had been previously noted, she remained well throughout this $3\frac{1}{2}$-year period. Her blood examination was entirely normal.

When seen in March 1963, there was no change in her status, but a marrow biopsy showed abnormal cells. She failed to attend for her next follow-up appointment, and inquiry revealed that she had died in June, 1963. As death had occurred outside the Hospital, no post-mortem had been performed and the cause of death was, therefore, unknown. The presence of abnormal cells (Burkitt tumour cells) in her bone marrow 3 months before her death would point strongly to this tumour as the cause of death.

This patient had survived for nearly 4 years, without chemotherapy, having had only a simple excision of the ovarian tumour.

### DISCUSSION

The clinical features and the histological appearances of the tumour in these 4 cases were considered typical of the African lymphoma (O'Conor and Davies, 1960). Tumour deposits in the breasts of 2 patients, although uncommon, can be expected in a neoplasm that is frequently widespread, and these deposits occurred after the more typical sites (ovaries and jaws) had been involved by the tumour.

Without any doubt, surgery does not normally play any part in the management of this disease. Excision of the ovarian masses in cases II and IV was carried out at a time when the true nature of the tumour was not appreciated. However, the period of apparent freedom from tumour of one, and nearly 4 years in cases II and IV respectively, would, in retrospect, support the action taken. The excision of the subsequent recurrence of tumour in the breast of case II was deemed necessary because a careful search had failed to reveal any other deposits and her bone marrow could not tolerate a further course of cyclophosphomide. This patient was free of tumour for 6 months following this.

Instances where surgical excision has been of any, albeit limited value, are rare. The majority of patients must, therefore, depend on chemotherapy. The repeated administration of mustine hydrochloride and cyclophosphomide contributed greatly to the survival of cases I, II and III. Since the regression of the tumour in the 3 patients occurred at a time when they were under treatment, the results achieved can be ascribed to the drugs used. It must be admitted, however, that these results could not be predicted nor have they been repeated in other patients similarly treated. Moreover, the survival of case IV for nearly 4 years without treatment other than simple excision of the ovarian mass is difficult to explain in the light of the known natural history of this neoplasm. It is probable therefore that other factors, at present unknown, may have played a part in the survival of these patients. The operation of an immunological agent cannot be ruled out in these cases and reports by Burkitt, Hutt and Wright (1964, personal communication) in which tumour regression occurred after treatment had been abandoned or was incomplete, would lend support to this concept. If such an agent were present, it would theoretically be easier to detect in patients who have survived longer than in those who died early in the disease. This calls for further investigation.

### SUMMARY

The case reports of 4 patients with the African lymphoma, or Burkitt tumour, are presented.

The survivals of 2½–4 years were considered longer than previously recorded in this disease. In three patients, this survival could be ascribed to the drugs used, but as no such drugs had been given to the fourth patient who had survived 4 years after the simple excision of an ovarian lymphoma, it is difficult to explain the survivals on the basis of the drugs alone. It is probable that other factors including a possible immunological factor play a part in these survivals.

I am most grateful to Professor Ogunlesi and Dr. Una Lister for permission regarding cases III and IV, and to the Departments of Morbid Anatomy and Medical Illustration for the photomicrographs and illustrations.

### REFERENCES

Burkitt, D.—(1958) *Brit. J. Surg.*, **46**, 218.—(1962) *Postgrad. med. J.*, **38**, 71.
O'Conor, G. T. and Davies, J. N. P.—(1960) *J. Pediat.*, **56**, 526.
Ottgen, H. F., Burkitt, D. and Burchenal, J. H.—(1963) *Cancer*, **16**, 616.
*Idem*, Clifford, P. and Burkitt, D.—(1963) *Cancer Chemother. Rep.*, **28**, 25.